# Development of a Novel Acoustic Spectroscopy Method for Detection of Eggshell Cracks

**DOI:** 10.3390/molecules26154693

**Published:** 2021-08-03

**Authors:** István Kertész, Viktória Zsom-Muha, Rebeka András, Ferenc Horváth, Csaba Németh, József Felföldi

**Affiliations:** 1 Institute of Food Science and Technology, Hungarian University of Agriculture and Life Sciences, 1118 Budapest, Hungary; Zsomne.Muha.Viktoria@uni-mate.hu (V.Z.-M.); andras.rebeka@gmail.com (R.A.); horvath.ferenc@spar.hu (F.H.); Felfoldi.Jozsef@uni-mate.hu (J.F.); 2 Capriovus Kft., 2317 Szigetcsép, Hungary; nemeth.csaba@capriovus.hu

**Keywords:** egg, eggshell, acoustics, crack detection, non-destructive testing, spectroscopy

## Abstract

Non-destructive testing (NDT) for eggshell faults is highly important for the egg industry, as cracked eggs account for around 3% of total production. The most commonly used method at present, candling, is labor intensive, while computer vision systems are expensive and complicated. In this paper, we present a simple, yet efficient, novel method for eggshell crack detection by acoustic spectroscopy. Altogether, 693 sound recordings were evaluated by different classification methods. The results show a cross-validated 2.1% total classification error, with only 0.87% false positive rate, which is the crucial metric for fresh eggs. Adapting the developed method to an industrial setting may lead to a reliable, fast and cost-effective detection method.

## 1. Introduction

Non-destructive testing (NDT) for eggshell and internal egg defects is very important for the industry for two reasons: 1. as opposed to other foodstuff, destructive testing of a batch sample cannot be extrapolated to critical faults of individual pieces, which account for about 3% of total production [1]; 2. quasi-non-destructive testing would completely null the value of the product for customers. These two factors created an ideal circumstance for researching NDT solutions for the industry. In addition, the most commonly used method at present, candling, requires extensive human labor [2,3].

There have been several attempts for automation of the eggshell crack detection process with the aid of computer vision systems, but these are very costly, and their reliability is questionable. Nevertheless, the pressure of the industry has led to creative solutions, such as the one described in a patent [4], which is able to detect microcracks but also requires a pressurized chamber of alternating pressures and, therefore, is expensive and difficult to implement. This shows the difficulties faced when high precision is a factor to consider. Classification of cracked eggs with computer vision systems has proven to be difficult: Wu et al. [5] managed to achieve only 93% validated correct classification using soft-margin support vector machine (SVM) classification.

Detection of cracks based on visual cues is difficult due to two main factors: computer vision systems can only see the samples in two dimensions, which means it is required to move the eggs in a calculable manner (direction- and speed-wise); detection of surface faults on constantly moving ovoid bodies with a high variance in spatial dimensions is an immensely complex task. This can also create a bottleneck for automated and operator-dependent solutions. Although candling can be used for internal defect detection or observation of embryos, these details are more pronounced at later stages, and the problem affecting consumer acceptance from the very beginning is the presence of shell defects. The other problem is that there ought to be no lower threshold on the size of the fault; it is a matter of presence or absence, meaning the tiniest cracks, in some cases with width measured in microns, need to be detected [6]. A great benefit of visual techniques is that they bear the possibility of applying spectroscopic analysis to measure internal qualities [7], which is difficult (but not impossible, as with estimation of density and mass (0.62–0.68) in Wang et al. [8]) with acoustic measurement.

The acoustic response is a feature that can be quickly determined and analyzed, and it requires less precision in an online industrial setting and significantly less data to be processed as opposed to multiple high-resolution pictures. These factors made it a prevalent technology in the industry, although it is not perfect. Coucke et al. [9] published a study in 1997 concerning successful testing for fertility of eggs based on acoustic signals but pointed out in their later study and doctoral thesis [10,11] that the acoustic response of the egg is mostly characterized by the mechanical properties of the shell. Their findings were verified by Attar and Fathi [12], who found a strong correlation (0.97) between resonance frequency and shell strength. Their method was further developed by De Ketelaere [13,14] and Cho et al. [15]. Cho’s team, using discriminant analysis, achieved 4% and 6% misclassification errors for intact and cracked eggs, respectively. Li et al. [16] applied the Bayesian probabilistic classifier on wavelet transforms of acoustic responses for the detection of cracks, reaching a correct classification rate over 95%, but a precise value was not stated. Jindal and Sirtham [2] reported the identification of 99% of all cracked eggs, but their false rejection rate was 10%. In contrast to this, Coucke’s method based on the dynamic stiffness (K_dyn_) calculated from the spectrum gave a false rejection of 1%, and it was used as the basis for later research by De Ketelaere [17] and Bain et al. [18]. Deng et al. [19] applied a wavelet transformation-based technique, reaching a maximum of a 98.9% detection rate with SVM and a 0.8% false rejection rate, but it was unvalidated, meaning the actual prediction capability of their model is undoubtedly worse.

Despite these issues, the most reliable method for crack detection on an industrial scale remains the acoustic response method. Even though it is the prevalent technique, as discussed in this section, there is still room for improvement, as the producers tend to heavily underestimate the ratio of defected eggs by approximately half [18].

## 2. Results

### 2.1. Performance Indicators

First, a general prediction was performed with all the settings discussed in the Materials and Methods section to provide an overall picture of which methods should be taken into consideration for later calculations. The tested discriminants were K-nearest neighbors (KNN), SVM, decision trees, linear discriminant analysis (LDA) and quadratic discriminant analysis (QDA). Results for the three performance indicators are shown in Table 1, Table 2 and Table 3 (no decimal precision was provided by the built-in application of MATLAB for false positives).

It is clear that window size contributed to the classification performance, but the multiplier value did not. The speed of classification shows very high values, but they decline rapidly with multiplier and window size. This indicator, although being important on an industrial scale, can be boosted easily by using the right hardware and firmware, and, therefore, it is considered less relevant than the other two.

Summing up all of the conclusions from the results presented in the tables, QDA and SVM classifiers showed the best performance, and the values of indicators were not consistent or poor for LDA and KNN. The performance difference between QDA and SVM becomes evident when the false positive rates are interpreted correctly: SVM misses the detection of a crack approximately one and a half times more often than QDA; that is, for two faulty eggs missed by QDA, there is a third missed by SVM. Therefore, in the future, only the QDA classifier should be used.

### 2.2. Classifier Eligibility Testing

Despite the obvious prevalence of QDA over LDA, it is important to evaluate whether QDA should be applied instead of LDA. The difference between LDA and QDA is that for QDA, the covariance matrices of variables between groups of observations are different, whereas for LDA, they are equal.

**Figure 1:**
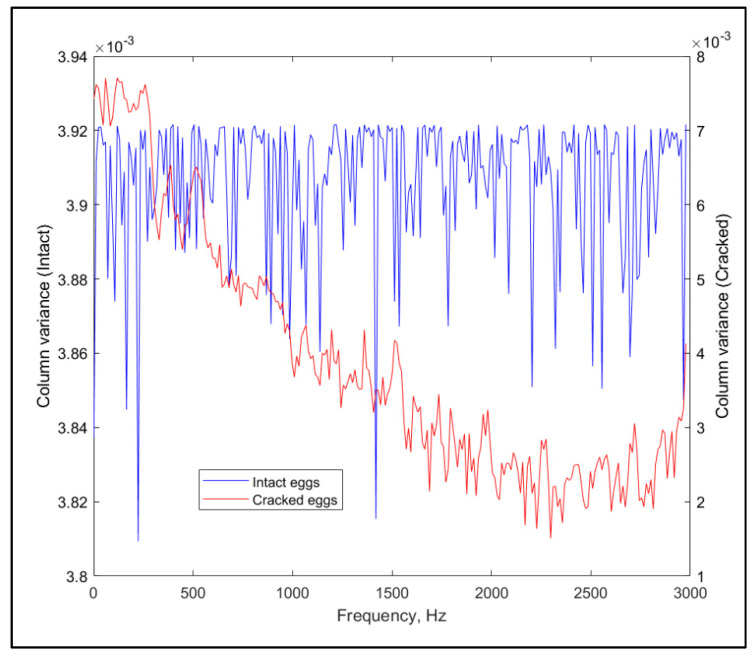
Column variances of the PCA coefficients for the two groups of samples.

In case inequality is a valid assumption, the difference between the spectra of the intact and cracked eggs should be captured in the principal components from principal component analysis (PCA). This can be easily verified by plotting the diagonal (containing the column variances) of the covariance matrix corresponding to the coefficients of the components with the highest variance explanation power (component number one) against the original variables for the two groups. Different covariance matrices manifest in different loading (coefficient) patterns, as shown in Figure 1 (for laid down position only).

**Figure 2:**
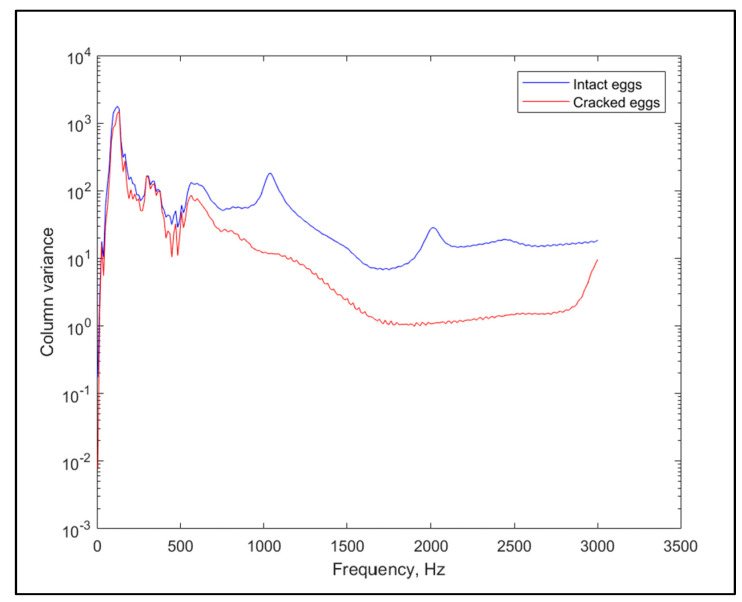
Column variances of the FFT coefficients for the two groups of samples.

Moreover, disparities in covariances of the original variables (i.e., the spectra) may give us insights into which frequencies may describe other frequencies well, revealing important regions for differences in the sounds. The main differences in curve shape (not only the height) can be found in the interval between 500 Hz and 3000 Hz as seen in Figure 2 for the laid down position.

### 2.3. Final Crack Detection Results

After verification of the applicability of QDA, the method should be rigorously tested while pushing the limits of the prediction performance, using the two most important indicators: total classification error and false positive detections of faulty eggs. A high fold number for cross-validation (20) was used, to ensure the validity of the results. A window size of 2^14^ and a multiplier of 2^1^ were used.

The number of latent variables was calculated by minimizing the total classification error, which corresponded with the false positive error rate. This ultimately resulted in a 2.1% prediction error for the total number of misclassifications and a 0.87% prediction error for false positives, as shown in Table 4, using 40 latent variables that accounted for the 99.6% explained variance.

## 3. Discussion

Egg crack detection resulted in high validation accuracy based only on the FFT spectrum magnitude values used as predictor variables. This suggests that it is not only the resonance frequency shift used in earlier studies that can reliably be used for prediction of faults, but the spectrum magnitudes themselves capture the important variations caused by physical changes in the shell.

The classification method proposed in this paper, based on acoustic excitation and FFT analysis of the eggs, was purposely tested against cracks that are very difficult and, in some cases, impossible to detect by candling. The 2.1% total misclassification and 0.87% false positive error rate achieved by our method, compared to many published in this field, are validated and are among the best-validated results found in the literature, showing promise for industrial application.

In an industrial setting, the most problematic aspects are external sources of noise and automation of a steady excitation process. The problem of noises can be addressed by using a tunnel with quasi-soundproof insulation. This can lower the medium- and high-pitch external noises considerably, and, at the same time, low-frequency vibrations of machines are not of concern, as they appear outside the bandwidth of interest, 500–30,000 Hz. The other important tool for proper signal extraction is the microphone. The use of a high-sensitivity lobar polar pattern condenser microphone is suggested, as the sensitivity pattern is ideal for focused, unidirectional sound capture, further decreasing the effects of ambient noise. Such microphones are relatively cheap (in the range of a few hundred euros), especially when compared to high-quality industrial cameras used for automated candling. This allows for speeding up of the grading process using multiple grading lines, as the costs of extension are much lower than those of candling. As a side note, as shown in Table 3, the calculation time for individual sample classification is extremely low, does not require high computation power and is completely automatic. This keeps the costs low, and the process will not increase the grading time. The other condition for a successful automation of the method is either conversion of the steady excitation protocol to a continuously applicable one, or the adaptation of the grading line by adding a stage for the egg coming to a rest. For the latter, as the procedure of excitation and recording requires less than one tenth of a second, they do not create a bottleneck, and a very short stoppage is sufficient. Furthermore, the laid down position favors the automation, since this is the natural rolling and resting position for eggs, and, therefore, no manipulation of the alignment is required. This results in a seemingly continuous procedure, as the whole grading process can be completed in less than one second, which is the lower end of the industry standard. The other option, excitation of rolling samples, can be carried out by stationary obstacles acting as exciters, which allows a completely continuous process. In this case, the time between the successive samples needs to be adjusted to maintain separation of the signals and synchronization of recording, but it opens up the possibility of even faster grading.

The supporting pad used in the experiment was made of a relatively stiff polyurethane foam and had an impermeable surface layer; therefore, it is applicable in industrial equipment with the possibility of cleaning and, if necessary, easy replacement. The main purpose of the foam is to minimize the conduction of ambient vibrations, and, therefore, other, nonrigid materials may be suitable for supporting the samples during the excitation if necessary.

## 4. Materials and Methods

**Figure 3:**
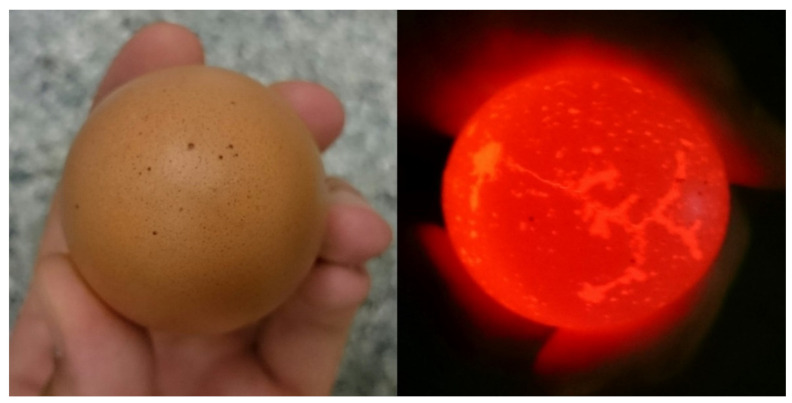
Seemingly intact egg (**left**) and cracks revealed on the same egg by a bright light shining through (**right**).

Acoustic measurements were conducted through a course of six weeks on medium-sized eggs (53–63 g). The restriction to medium size was applied because this is the most common size in the industry, especially for processed products, and the aim of the study was to prove that the principles of the present method can be applied. Furthermore, as preliminary infinite-element modelling has shown, size does not create profound changes in the resonance spectra of the eggshells. Samples were stored at 22 °C (± 1 °C), between 50–70% relative humidity. The storage experiment was set up in order to follow mass loss and other structural and chemical changes, but this part of the experiment was unsuccessful. Because of this, the hereby described measurements do not address the storage effect. Before starting the measurements, all samples were tested for faults by candling; a bright light was shone through the eggs to reveal hair cracks (see Figure 3).

All eggs were measured on the zeroth day, and 20 eggs were weekly tested for constituents, which meant cracking them open in the process; therefore, these samples were removed from the set. Before opening them completely, a very small (most of the time invisible) thin microcrack was inflicted on the pointy tip (opposite the air chamber) of the eggs, and acoustic testing was conducted before and after this destructive action, totaling 705 measurements over six weeks. The test itself was carried out by excitation with a hollow metal rod, meaning a single light knock on the shell. This knock was performed in both an upright (north–south, NS) and a laid down (east–west, EW) position on the uppermost part, meaning the pointy tip in NS and along the equatorial circumference in the EW arrangement. For stabilization and noise reduction, in both positions, the samples were placed on a foam pad (as suggested by Felföldi and Ignát [20]) hollowed out for the insertion of a microphone.

**Figure 4:**
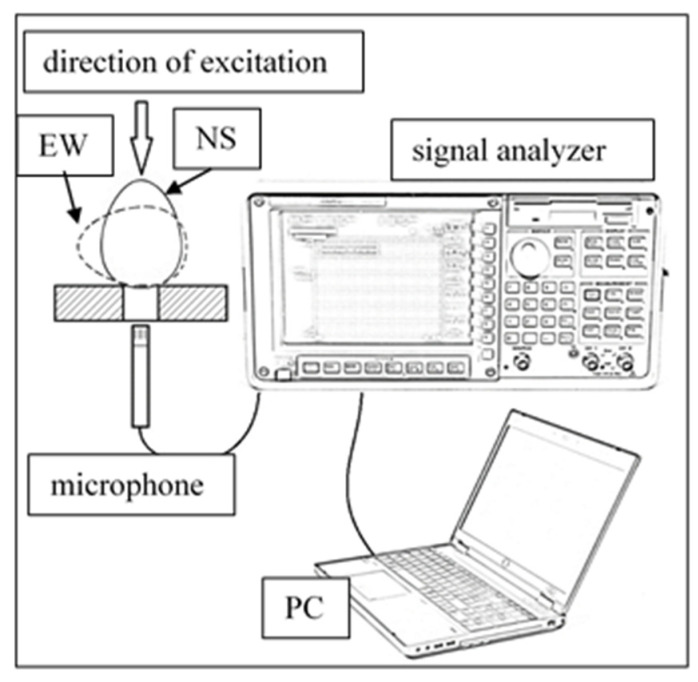
Experimental setup for the acoustic measurement of eggs in two positions.

For recording, a very sensitive microphone was used with a practically flat spectrum sensitivity. This instrument was connected to a Hewlett-Packard 53670A signal analyzer, from which the sound signal was sent to a laptop for recording with 96,000 Hz sampling frequency in lossless wav format. The experimental setup is shown in Figure 4. The sound files were processed with a program compiled in the MATLAB 2017a environment.

**Figure 5:**
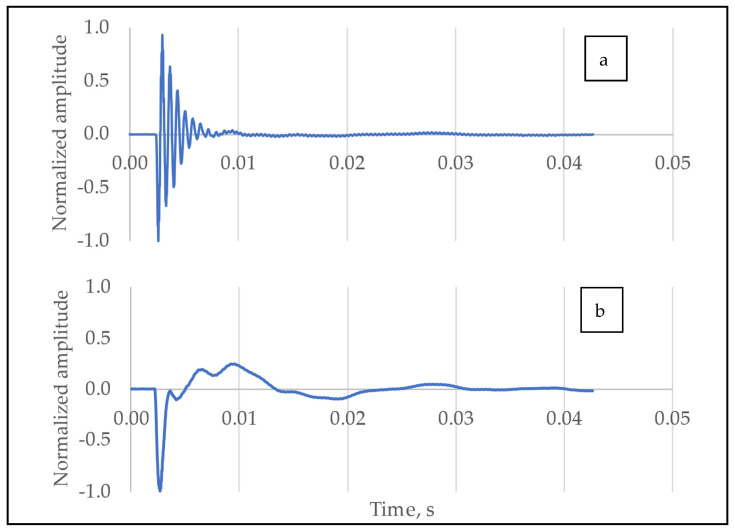
Normalized response signals of the eggs in NS (**a**) and EW (**b**) positions.

After removing erroneous measurement files, the total number of processed measurements was 693. The signals were first cropped to length dictated by the desired window size (but no less than 4096 points), which includes the most information-rich part of the response signals. The window size of the Fourier transform is defined as the length of the part (or the entirety) of the signal, measured in the number of data points, that is being converted by the transformation process into a spectrum for representation on the frequency domain. The signals were grouped according to the position of testing; examples for the two signals are shown in Figure 5.

**Figure 6:**
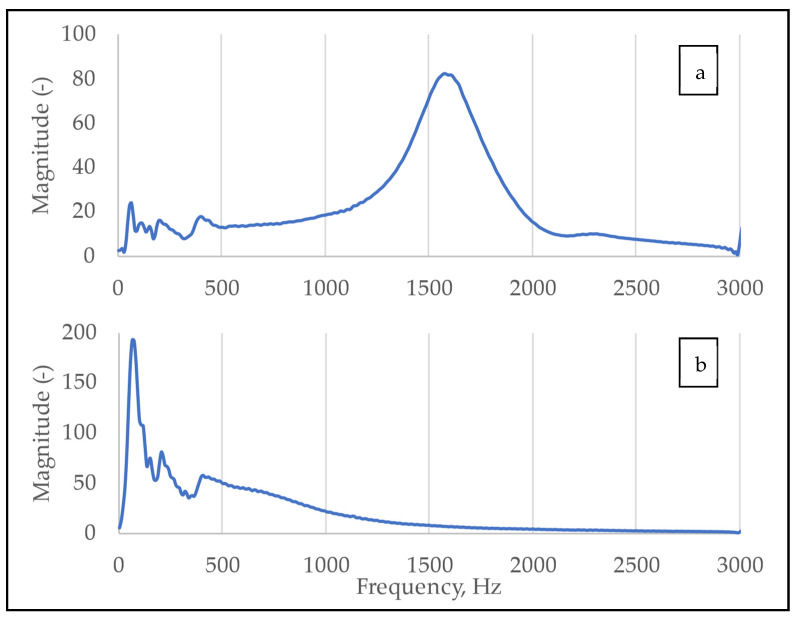
Spectra of the signals in NS (**a**) and EW (**b**) positions.

After normalization, fast Fourier transforms (FFT) of the signals were calculated with different window sizes to assess the effect of increasing resolution. Examples of the spectra of the response signals in the two positions are shown in Figure 6.

As an extra step, padding with zeros was also carried out, functioning as a basis to use multipliers for further increase in the resolution. The number of padding zeros was calculated as

Z = N(M − 1) (1)

where N is the number of data points in the signal, and M is the value of the multiplier. These multipliers take the form of the powers of two, because the FFT algorithm window sizes are also restricted to 2^n^ points. The lowest number used as the multiplier was 2, meaning the total length of the padded signal was double the original. The applied window sizes were not necessarily matched, resulting in the original, lower resolution with a running window, theoretically capturing more of the variation in time.

This manipulation of the resolution was carried out in order to assess whether more meaningful information can be extracted from the spectrum than what can be extracted using default window sizes. This is a useful technique when the precise locations of moving peaks (searching for resonant frequencies) are to be identified. However, it might also have an effect on using the magnitude values as variables, because with relocation of resonant frequency peaks, magnitude change in their original location occurs. This effect can also stack along neighboring frequencies and can be exploited if the entire spectrum is used as a series of explanatory variables.

Spectra were considered where 0 Hz < *f* < 3000 Hz, because there was no apparent change apart from the noise in the higher frequencies; the lowest frequency was determined by the resolution. The magnitudes of all frequencies were used for classification, but for NS and EW positions separately, because on an industrial scale, it is not realistic to expect that sorting machines can move around eggs and handle both positions in a reasonable timeframe.

In all cases, the spectrum was first processed with PCA to ensure the independence of the variables, which is a pivotal assumption for some of the classifiers tested. Principal components were calculated up to 99% of the described variance in the original data (i.e., the spectrum magnitudes).

For classification, a range of techniques were tested, including linear and nonlinear prediction methods: KNN, SVM, decision trees, LDA and QDA. Among the metrics for performance were the total number of misclassifications, the number of false positives for cracked eggs (as the most important aspect in the industry) and the speed of classification for new independent samples, which obviously depends greatly on the computer used for estimation but is a good basis for comparison between different methods.

All classification techniques were tested on spectra gained with window sizes of 2^12^, 2^13^ and 2^14^ and multipliers of 2^1^, 2^2^ and 2^3^, giving nine results per performance indicator per classifier per position (a total of 216). The result for the NS positions were subpar; therefore, they were omitted from further analysis. In all cases, K-fold cross-validation with 20 folds was applied to avoid underestimation of falsely classified samples.

## Figures and Tables

**Table 1:** Group prediction accuracy (%) of different classification methods.

Window Size			2^12^			2^13^			2^14^	
Multiplier		2^1^	2^2^	2^3^	2^1^	2^2^	2^3^	2^1^	2^2^	2^3^
**Classifier**	LDA	94.8	94.2	94.5	94.0	94.6	94.6	94.6	95.0	94.7
	QDA	95.5	95.0	94.3	96.3	95.6	95.6	97.2	96.2	97.0
	SVM	95.2	95.9	95.2	95.9	95.3	95.6	96.3	96.3	96.0
	KNN	93.2	92.8	93.3	94.7	94.2	94.9	94.9	94.9	94.7

**Table 2:** False positives for crack presence (%) of different classification methods.

Window Size			2^12^			2^13^			2^14^	
Multiplier		2^1^	2^2^	2^3^	2^1^	2^2^	2^3^	2^1^	2^2^	2^3^
**Classifier**	LDA	6	6	6	6	6	6	6	5	6
	QDA	3	3	3	3	3	3	2	3	2
	SVM	5	4	5	3	5	4	3	3	3
	KNN	6	6	6	5	5	4	4	5	5

**Table 3:** Speed of classification (s^−1^) of different classification methods.

Window Size			2^12^			2^13^			2^14^	
Multiplier		2^1^	2^2^	2^3^	2^1^	2^2^	2^3^	2^1^	2^2^	2^3^
**Classifier**	LDA	610	450	130	430	250	220	170	160	91
	QDA	610	500	270	410	210	230	210	100	92
	SVM	780	270	140	480	220	270	260	150	95
	KNN	750	380	140	470	270	260	260	100	91

**Table 4:** Validated classification matrix of the optimized QDA algorithm.

		True	
		Intact	Cracked
predicted	intact	566	6
	cracked	9	112

## Data Availability

The data presented in this study are available on request from the corresponding author. The data are not publicly available due to the sizeable raw data files requiring storage on an offline hard drive for safekeeping.

## Sample Availability

Samples are not available due to being perishable goods.
